# The effect of intrapartum prolonged oxygen exposure on fetal metabolic status: secondary analysis from a randomized controlled trial

**DOI:** 10.3389/fendo.2023.1204956

**Published:** 2023-06-27

**Authors:** Fang Chuai, Tong Dong, Yuan Liu, Wen Jiang, Lanmei Zhang, Lei Chen, Yunhai Chuai, Yuhang Zhou

**Affiliations:** ^1^ Department of Obstetrics and Gynaecology, Sixth Medical Center, Chinese PLA General Hospital, Beijing, China; ^2^ Department of Obstetrics and Gynaecology, Seventh Medical Center, Chinese PLA General Hospital, Beijing, China; ^3^ Department of Obstetrics and Gynecology, PLA Strategic Support Force Characteristic Medical Center, Beijing, China; ^4^ Department of Day Treatment, Sixth Medical Center, Chinese PLA General Hospital, Beijing, China

**Keywords:** prolonged oxygen exposure, umbilical cord arterial metabolites, fetal heart rate tracings, pregnancy, childbirth

## Abstract

**Objective:**

The aim of the study is to assess the effect of maternal prolonged oxygen exposure during labor on fetal acid–base status, fetal heart rate tracings, and umbilical cord arterial metabolites.

**Design:**

The study was conducted as a secondary analysis.

**Setting(s):**

The study was set in three tertiary teaching hospitals in Beijing, China.

**Participants:**

Approximately 140 women in the latent phase of labor with no complications participated in the study.

**Intervention:**

Participants were randomly allocated in a 1:1 ratio to receive either 10 L of oxygen per minute in a tight-fitting simple facemask until delivery or room air only.

**Main outcome measures:**

The primary outcome was the umbilical cord arterial lactate.

**Results:**

Baseline demographics and labor outcomes were similar between the oxygen and room air groups; the time from randomization to delivery was 322 ± 147 min. There were no differences between the two groups in the umbilical cord arterial lactate (mean difference 0.3 mmol/L, 95% confidence interval −0.2 to 0.9), the number of participants with high-risk category II fetal heart rate tracings (relative risk 0.94, 95% confidence interval 0.68 to 1.32), or the duration of those high-risk tracings (mean difference 3.6 min, 95% confidence interval −9.3 to 16.4). Prolonged oxygen exposure significantly altered 91 umbilical cord arterial metabolites, and these alterations did not appear to be related to oxidative stress.

**Conclusion:**

Maternal prolonged oxygen exposure during labor did not affect either the umbilical cord arterial lactate or high-risk category II fetal heart rate tracings but might result in alterations to the umbilical cord arterial metabolic profile.

**Clinical trial registration:**

www.clinicaltrials.gov, identifier NCT03764696.

## Background

Maternal oxygen (O_2_) administration was approved for use in preventing or treating fetal hypoxia and acidemia in many parts of the world (1–3). Obstetricians and midwives hoped that the supplemental O_2_ could be transferred to fetal circulation to improve fetal metabolic status and alleviate non-reassuring fetal status, as a mass of animal and human data have shown that maternal O_2_ improves fetal oxygenation and other neonatal outcomes (1–3). It is estimated that more than half of women during labor receive supplemental O_2_, even though they are well oxygenated (1). The duration of O_2_ exposure was often several minutes and sometimes hours, with no guideline or consensus; there was also no standard for the concentration of inhaled O_2_. However, in randomized clinical trials (RCTs), intrapartum O_2_ administration did not seem to increase fetal O_2_ content, either at a low or high fraction of inspired O_2_ (FiO_2_) [30% in Qian et al. (4), 80% in Thorp et al. (5), and Raghuraman et al. (6)], or for a short time or long time [minutes in Moors et al. (7) to hours in Chuai et al. (8)] (4–12). Two of those RCTs [Thorp et al. (5) and Chuai et al. (8)] found that 80% FiO_2_ administration resulted in a deterioration of the umbilical cord arterial (Ua) pH at birth, but four other trials [Sirimai et al. (9), 30% in Qian et al. (4), 80% in Raghuraman et al. (6) and Moors et al. (7)] did not find that O_2_ can affect the Ua pH (4–12); one of those RCTs [Moors et al. (7)] found that 80% FiO_2_ inhalation improved the fetal heart rate (FHR) tracings, but three other trials [30% in Qian et al. (4), 80% in Raghuraman et al. (6), and Chuai et al. (8)] did not find this improvement (4–12). It is difficult to determine whether intrapartum O_2_ exposure is beneficial or potentially harmful. No certain conclusions can be drawn due to the inconsistent results regarding fetal outcomes.

Our recent randomized trial found useless and harmful results for the effect of intrapartum prolonged O_2_ (60% to 80% FiO_2_) exposure on the umbilical cord venous (Uv) partial pressure of O_2_ (PO_2_) and Ua pH, prolonged O_2_ did not increase the Uv PO_2,_ but was associated with lower Ua pH (median 7.23 vs 7.27) compared with room air (8). Our result was questionable because the low Ua pH events (<7.2) were recorded to be the same between the O_2_ and room air groups, and there was also no time-dependent effect of O_2_ exposure on Ua pH (8). It was an unexpected result that the prolonged high degree of O_2_ administration did not change the fetal O_2_ content or acid–base status significantly. We performed this secondary analysis of data and samples from this trial with the objective of further investigating the effect of prolonged O_2_ exposure on fetal metabolic status, including Ua lactate, which was a more sensitive and specific marker than Ua pH for predicting metabolic acidosis and short-term newborn morbidity, and Ua metabolomics, which could be a powerful approach to studying the molecular mechanisms and metabolic pathways in response to supraphysiological O_2_ (13, 14).

## Methods

### Study design

We conducted a secondary analysis of a randomized trial in which women with category I FHR tracings in the latent phase of labor were assigned 10 L per minute of O_2_, or room air. The trial was registered on ClinicalTrials.gov with the identifier NCT03764696 and conducted at three tertiary teaching hospitals between January 2021 and October 2021 (8). Approval from the ethics committees and informed consent from all participants were obtained. The registered study protocol is available in **Supplementary Material 1**.

### Study population, randomization, and intervention

The trial included at term (37 to 42 weeks), singleton, cephalic presentation pregnant women with category I FHR tracings in the latent phase of labor and excluded participants with existing medical or obstetric complications, including respiratory or cardiovascular disease, diabetes mellitus or insulin-treated gestational diabetes mellitus, hypertension, or preeclampsia, cephalopelvic disproportion, oligohydramnios, fetal growth restriction, anemia, fever, tobacco, or alcohol use, etc.

At the point in the latent phase of labor (2 to 3 cm of cervical dilation in nulliparity and 1 to 2 cm of cervical dilation in multipara), participants were assigned 1:1 to receive either O_2_ via the tight-fitting simple facemask at a flow rate of 10 L per minute (60 to 80% FiO_2_) or room air only without a facemask. The facemask was checked by research nurses to ensure that it covered the nose and mouth during labor. The two interventions (O_2_ vs room air) were continued until delivery.

All women received standard intrapartum care and began pushing down immediately at the onset of the second stage in a supine position. The electronic fetal monitoring was reviewed every 15 to 30 min in the first stage and continuously in the second stage of labor (15). The interpretation of FHR tracings and the methods of intrauterine resuscitation followed the guidelines of the American College of Obstetricians and Gynecologists (ACOG) (15).

### Outcomes and data collection

The primary outcome of this analysis was the Ua lactate. The paired Uv and Ua blood samples were collected as recommended by ACOG (16), and these samples (150 μl per sample) were analyzed using the Gem Premier 4000 benchtop blood gas analyzer (Werfen America). We considered gases to be valid if the Uv–Ua pH difference was >0.02, the Ua–Uv partial pressure of carbon dioxide (PCO_2_) was >5.25 mmHg, and the Uv PCO_2_ was >21.75 mmHg (17). Only women with paired and validated Uv and Ua blood gases were included.

The secondary outcome was the high-risk category II FHR tracings, including the number of women who developed high-risk category II FHR tracings during labor and the duration of those tracings. The high-risk category II FHR tracings were defined as any of the following features: baseline bradycardia, minimal or absent variability, recurrent variable decelerations, and recurrent late decelerations, as these features might suggest an increased risk for fetal hypoxia or acidemia (12). Two trained research nurses, blinded to allocation and outcomes, assessed the FHR tracings independently and resolved disagreements by involving a third nurse. Another secondary outcome was the Ua metabolite analysis. The Ua blood samples (500 μl per sample) were obtained immediately after delivery and separated by centrifugation at 3,000 rpm for 10 min at 4 °C. Plasma samples were stored immediately at −80 °C and transported on dry ice for plasma ultra-high performance liquid chromatography-tandem mass spectrometry (UHPLC-MS/MS) analysis, which was performed by a commercial company (Novogene Co. Ltd., China). The experimental protocol is available in **Supplementary Material 2**. The rest of the plasma samples were used for testing the Ua malondialdehyde (MDA), which was assessed using a commercial assay kit (Beyotime China). Other outcomes were other umbilical cord blood sample components, including Uv/Ua glucose, K^+^, Na^+^, Cl^−^, and Ca^2+^, which were assessed using the blood gas analyzer (Werfen America).

### Statistical analysis

We used a fixed sample size from the primary trial, and all randomized participants were included in this analysis. Data analysis was performed using the modified intention-to-treat principle. All randomized women were included in the high-risk category II FHR tracing analysis; 96.4% (135/140) participants with validated paired Uv and Ua gases were included in the Ua lactate analysis; and 85% (119/140) participants were included in the Ua metabolites analysis. The Kolmogorov–Smirnov test was used to analyze the distribution of continuous variables. Baseline characteristics and outcomes were compared between the two groups using the Student’s *t* test or Mann–Whitney *U* test for continuous variables and the Chi-square test or Fisher exact test for categorical variables. The plasma UHPLC-MS/MS analyses included metabolite annotation, principal components analysis (PCA), partial least squares discriminant analysis (PLS-DA), differential metabolite identification, metabolic pathway enrichment, etc. The method of UHPLC-MS/MS analysis is available in **Supplementary Material 2**.

## Results

Of the 140 randomized participants, 70 received O_2_ and 70 received room air without interruption or crossover; 135 women were included in the Uv/Ua lactate analysis; all 140 women were included in the high-risk category II FHR tracing analysis; and 119 women were included in the Ua metabolite analysis. Baseline demographics and labor outcomes were similar between the O_2_ and room air groups. Most labor outcomes except Ua pH were similar between the two groups; the cesarean delivery rate was less than 3% in the trial; and the median duration of the first and second stages of labor was about 8 h (**Table 1**). In the O_2_ group, 94% of women received O_2_ for more than 2 h and 88% received more than 3 h, and the Ua pH was significantly lower in the O_2_ group than in the room air group (**Table 1**).

**Table 1:** Maternal demographics and labor outcomes.

Characteristics	Oxygen (n = 70)	Room Air (n = 70)	*P*
Maternal demographics			
Age, year	31.6 ± 3.6	31.6 ± 3.8	1.000
Gestational age, week	39.8 ± 1.0	39.7 ± 0.9	0.386
Nulliparity (%)	59 (84.3)	55 (78.6)	0.385
Admission body mass index, kg/m^2^	26.0 ± 2.6	25.8 ± 2.6	0.609
Labor outcomes			
Time from randomization to delivery, min	322 ± 147	308 ± 125	0.537
Cesarean delivery (%)	3 (4.3)	1 (1.4)	0.612
Female neonatal sex (%)	34 (48.6)	33 (47.1)	0.866
Birth weight, g	3315 ± 335	3416 ± 402	0.107
Neonatal resuscitation (%)	6 (8.6)	4 (5.7)	0.512
Umbilical arterial pH*	7.23 (7.20–7.27)	7.27 (7.20–7.30)	0.005
Category II fetal heart rate tracings (%)	57 (81.4)	55 (78.6)	0.672

Data are expressed as mean ± SD, number (%), or median (interquartile range).

*Samples for blood gas analysis: Oxygen group (n = 67), Room Air group (n = 68).

Data from Reference (8).

There were no differences between the O_2_ and room air groups in the Ua lactate (MD 0.3 mmol/L, 95% CI −0.2 to 0.9; *p* = 0.224), the Uv lactate (MD 0.3 mmol/L, 95% CI −0.2 to 0.8; *p* = 0.263); in the Ua glucose (MD 0.2 mmol/L, 95% CI −0.2 to 0.6; *p* = 0.263), the Uv glucose (MD 0.1 mmol/L, 95% CI −0.3 to 0.5; *p* = 0.547); or in some electrolytes, including Uv/Ua K^+^, Na^+^, Cl^—^, or Ca^2+^ (**Table 2**).

**Table 2:** Paired umbilical cord venous and arterial blood lactate, glucose, and electrolytes.

Outcomes	Oxygen (n = 67)	Room Air (n = 68)	*P*	MD	95% CI
Umbilical venous (Uv)					
Uv lactate, mmol/L	3.9 ± 1.6	3.6 ± 1.3	0.263	0.3	−0.2 to 0.8
Uv glucose, mmol/L	5.6 ± 1.1	5.5 ± 1.1	0.547	0.1	−0.3 to 0.5
Uv K^+^, mmol/L	4.4 (4.3–4.8)	4.4 (4.2–4.7)	0.381	–	–
Uv Na^+^, mmol/L	134 (133–136)	134 (133–135)	0.439	–	–
Uv Cl^—^, mmol/L	104.6 ± 1.8	104 ± 2	0.066	0.6	−0.04 to 1.3
Uv Ca^2+^, mmol/L	1.47 ± 0.08	1.48 ± 0.08	0.587	−0.01	−0.03 to 0.02
Umbilical arterial (Ua)					
Ua lactate, mmol/L	4.3 ± 1.8	4.0 ± 1.4	0.224	0.3	−0.2 to 0.9
Ua glucose, mmol/L	5.0 ± 1.1	4.8 ± 1.1	0.263	0.2	−0.2 to 0.6
Ua K^+^, mmol/L	4.3 (4.1–4.6)	4.2 (4.1–4.5)	0.14	–	–
Ua Na^+^, mmol/L	134 (133–136)	134 (133–135)	0.469	–	–
Ua Cl^—^, mmol/L	103.5 ± 1.9	103.1 ± 1.9	0.172	0.4	−0.2 to 1.1
Ua Ca^2+^, mmol/L	1.46 (1.4–1.5)	1.46 (1.43–1.49)	0.568	–	–

Data are expressed as median (interquartile range) or mean ± SD.

MD, mean difference; CI, confidence interval.

The rate of composite high-risk category II FHR tracings was similar between O_2_ and room air groups (48.6% vs 51.4%; RR 0.94, 95% CI 0.68 to 1.32; *p* = 0.735), and there were no differences between the two groups for the individual components of the composite, including baseline bradycardia, minimal or absent variability, recurrent variable decelerations, or recurrent late decelerations (**Table 3**). The total duration of high-risk category II FHR tracings in women was similar between O_2_ and room air groups (MD 3.6 minutes, 95% CI −9.3 to 16.4; *p* = 0.582) (**Table 3**).

**Table 3:** The rate and duration of high-risk category ii fetal heart rate (FHR) tracings.

Outcome	Oxygen (n = 70)	Room Air (n = 70)	*P*	MD or RR	95% CI
**Composite high-risk category II**	34 (48.6%)	36 (51.4%)	0.735	0.94	0.68 to 1.32
Baseline bradycardia	0 (0%)	1 (1.4%)	0.50	0.33	0.01 to 8.04
Minimal or absent variability	9 (12.9%)	8 (11.4)	0.796	1.13	0.46 to 2.75
Recurrent variable decelerations	24 (34.3%)	26 (37.1%)	0.724	0.92	0.59 to 1.44
Recurrent late decelerations	10 (14.3%)	11 (15.7)	0.813	0.9	0.4 to 2.0
Duration of high-risk category II*	58.8 ± 28.5	55.2 ± 25.5	0.582	3.6	−9.3 to 16.4

Data are expressed as number (%) or mean ± SD.

RR, relative risk; CI, confidence interval.

Minimal or absent variability: lasting 40 min or more.

Recurrent decelerations: more than 50% of uterine contractions were accompanied by decelerations for 20 min or more.

*Mean minutes of oxygen group (n = 34) and room air group (n = 36).

**Figure 1:**
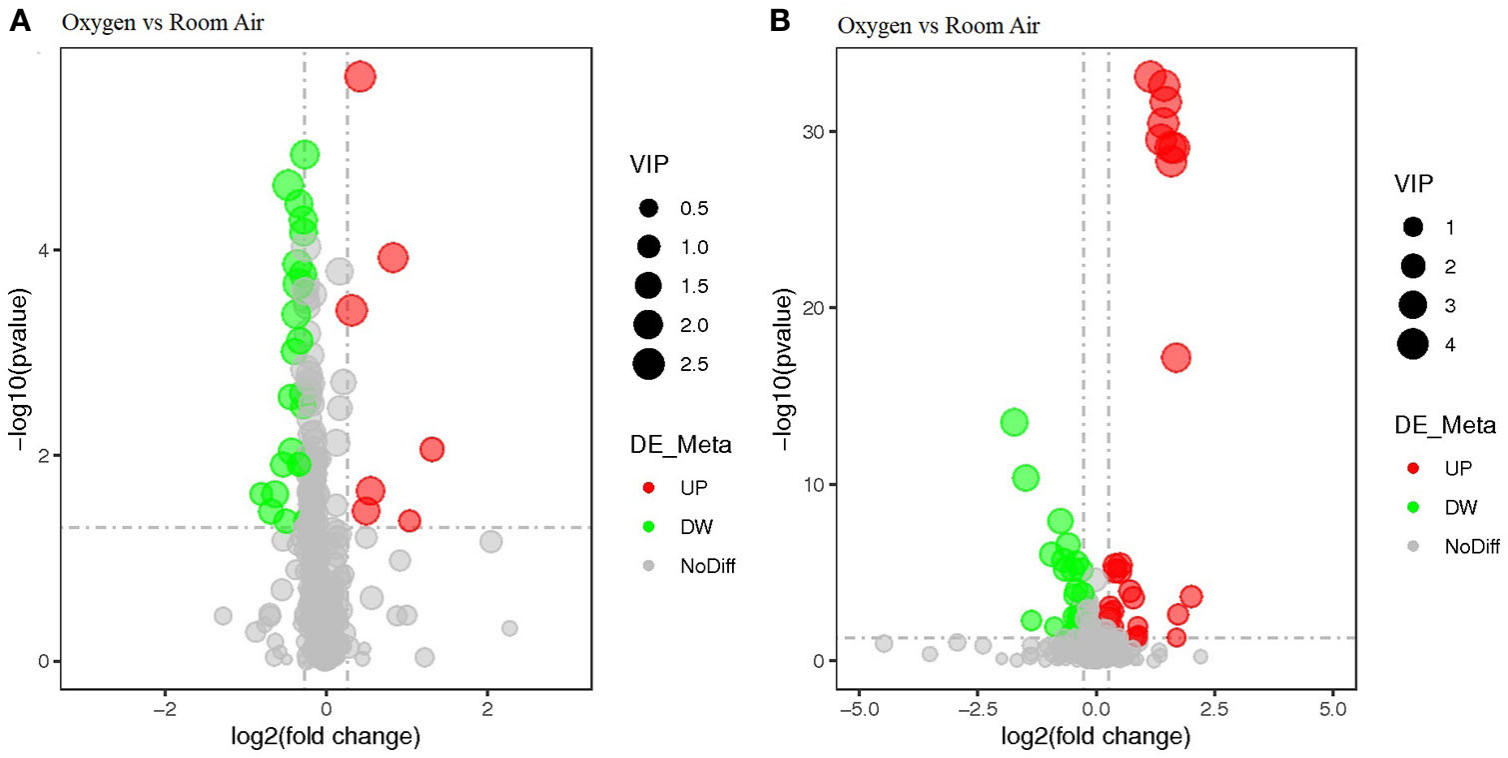
The volcano plot analysis of differential metabolites. Negative ionization mode **(A)** and positive ionization mode **(B)**.

**Figure 2:**
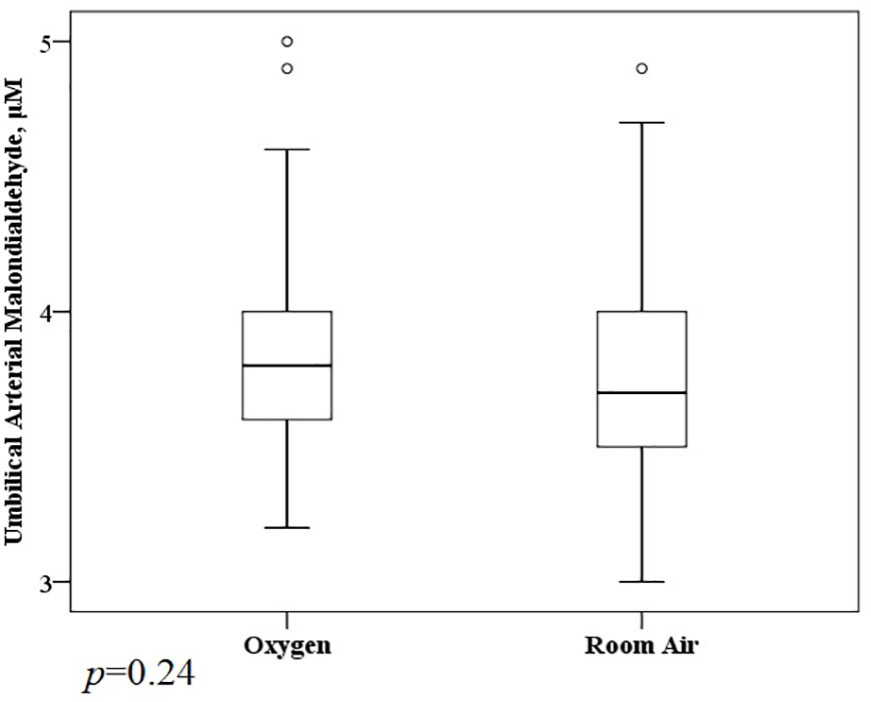
Median umbilical cord arterial malondialdehyde in women randomized to oxygen and room air.

A total of 1,117 molecular features with a weight of 100 to 1,000 Da were extracted (611 in positive mode, 456 in negative mode) (**Supplementary Material 3**). Prolonged O_2_ exposure during labor significantly altered 91 metabolites (38 showed a remarkable increase and 53 were reduced significantly) in the Ua plasma (**Figure 1**). The KEGG enrichment analysis found some metabolic or signaling pathways, including tryptophan metabolism, circadian entrainment, riboflavin metabolism, folate biosynthesis, RNA transport, the Ras signaling pathway, the Rap1 signaling pathway, the sulfur relay system, and endocytosis (**Supplementary Material 3**). However, we failed to find that O_2_ could directly affect these differential metabolites or enrich metabolic or signaling pathways. There was no difference between the O_2_ and room air groups in the Ua MDA (**Figure 2**).

## Discussion

### Main findings

The present trial was a negative study; it showed that prolonged O_2_ exposure did not affect fetal acid–base status (Ua lactate) or relieve high-risk category II FHR tracings in normal women during labor. Prolonged O_2_ exposure significantly altered some Ua metabolites; however, these alterations were not associated with a unique metabolic or signaling pathway and did not appear to be related to oxidative stress.

### Interpretation

To our knowledge, this is the first randomized trial about intrapartum prolonged O_2_ exposure (mean 322 min), in contrast to previous studies including Thorp et al. (5) (mean 45 min), Qian et al. (4) (median 50 min), Raghuraman et al. (6) (median 96 min), and Moors et al. (7) (median 12 min). At the long-duration, high-concentration level of O_2_, we did not find a definite harmful effect on fetal Ua lactate, which was more sensitive and specific than Ua pH; however, we still could not demonstrate any benefit in improving FHR tracings. At present, there are only six RCTs that addressed maternal O_2_ administration during labor; most of the trials were negative studies that failed to prove that O_2_ was superior to room air (4–12). Only two studies reported that O_2_ was associated with lower Ua pH or more Ua pH less than 7.2 events (5, 8), and one study showed O_2_ relieved suspicious or abnormal FHR tracings (7). Recent systematic reviews with moderate heterogeneity showed no association between maternal O_2_ administration and a clinically relevant improvement in Ua pH or other fetal outcomes during labor or a scheduled caesarean section (18, 19).

As most of the previous studies were negative, we used a more discriminating metabolomic approach in this secondary analysis. We found 38 metabolites showed a remarkable increase, and 53 metabolites were significantly reduced in the Ua plasma after prolonged O_2_ exposure. The primarily enriched pathways were not unique; they were tryptophan metabolism, riboflavin metabolism, folate biosynthesis, RNA transport, the Ras signaling pathway, the Rap1 signaling pathway, etc. However, O_2_ might not be directly related to these 91 differential metabolites or enriched metabolic or signaling pathways. Theoretically, prolonged O_2_ exposure may lead to increased maternal and fetal free radical activity (18, 19). In this analysis, these metabolites (100 to 1,000 Da) or pathways did not appear to be related to oxidative stress, and there was also no difference between the O_2_ and room air groups in the Ua MDA (72 Da), which was the most studied marker in trials. It is thought that hyperoxia can cause maternal oxidative stress, which is controversial in the fetus. The recent systematic review showed O_2_ was associated with an increase in maternal MDA level but no significant difference in the Ua MDA level (18).

The theoretical basis of intrapartum O_2_ inhalation was that O_2_ supplementation might be helpful in improving fetal O_2_ content (8, 11). Generally, maternal arterial PO_2_ is about 100 to 110 mmHg, and it will drop to 40 to 50 mmHg in the intervillous space (20). O_2_ can enter the fetus through the placental barrier (vasculo-syncytial membrane, VSM) by simple diffusion, and fetal Uv PO_2_ is about 28 mmHg (20). Previous studies showed that five minutes of breathing 50% O_2_ increased maternal arterial PO_2_ over 200 mmHg and breathing 100% O_2_ increased it over 300 mmHg (21). Numerous animal and human studies have demonstrated that maternal O_2_ administration leads to an increase in fetal oxygenation and amelioration of abnormal FHR patterns (1). In non-human primates, James et al. (22) indicated that fetal hypoxia was the major cause of late deceleration, which could be addressed by increasing fetal PO_2_. In patients with non-reassuring FHR patterns during labor, Haydon et al. (23) showed that O_2_ inhalation (40 and 100% FiO_2_) increased fetal O_2_ saturation substantially, as determined by fetal pulse oximetry. A Cochrane Review reported a very low quality of evidence that women receiving supplementary O_2_ had a higher mean UvPO_2_ than participants who received room air for cesarean section during regional anesthesia (19). However, none of the RCTs so far have found that maternal O_2_ administration can increase fetal O_2_ content, neither at low nor high concentrations of O_2_ nor for a short or long period of O_2_ exposure. Maternal arterial blood has a limited capacity to carry O_2._ When maternal PO_2_ is over 150 mmHg, the hemoglobin will be saturated, and excess O_2_ can only be transported by an inefficient mode of transportation, physical dissolution (0.03 ml O_2_ per 1 mmHg) (24, 25). In addition, O_2_ overdoses have been found to cause maternal adverse events, including cardiac hemodynamics and oxidative stress (26, 27). In clinical practice, the current widespread use of O_2_ should be limited, especially in nonhypoxemic pregnant women. We do not seek to reach a conclusive recommendation regarding O_2_ exposure due to the heterogeneity of the study results. For future research, although most RCTs are negative studies, it is inconceivable that supra-physiological O_2_ would not affect the maternal–fetal interface. Understanding the cellular and molecular alterations in the VSM may improve our understanding of maternal–fetal material exchange.

### Limitations

This RCT has several important limitations. First, the sample size of the trial was small; the study included 140 participants and found no differences between the two groups in the Ua lactate (MD 0.3 mmol/L, 95% CI −0.2 to 0.9). To detect the 0.3 mmol/L difference in Ua lactate with 80% power and a 2-sided test of 0.05, we estimated that 450 women were needed in each group. Second, the main outcomes were not patient-relevant end points (28). The Ua lactate was only a laboratory finding, which was an effective way to measure fetal acidosis but limited in the prediction of hypoxic ischemic encephalopathy (HIE) (13). The intrapartum FHR monitoring was a subjective test with poor interobserver reliability (15). To detect the difference in HIE, about 11,000 participants would be required. Third, the results of the Ua metabolite analysis were questionable. Although some differential metabolites were identified between the two groups, we did not find that O_2_ could directly affect these metabolites through a literature search. The KEGG enrichment analysis revealed some potential metabolic or signaling pathways, but these pathways were not unique or unrelated to each other. The results might suggest that maternal hyperoxia had little effect on fetal metabolic status or that this analysis of metabonomics was spurious due to the heterogeneity of the small sample size. Fourth, the applicability of our study was low; the trial focused on changes in normoxia with further O_2_ administration but did not consider the effects of administering hyperoxia under hypoxic conditions. Maternal O_2_ during labor was usually administered to the fetus under hypoxic conditions; however, this trial did not include any high-risk fetuses with intrauterine asphyxia, abnormal FHR tracings, fetal growth restriction, etc.

## Conclusions

We conclude that prolonged intrapartum O_2_ exposure in normal labor does not affect either the Ua lactate or high-risk category II FHR tracings. This way of administering O_2_ might result in alterations to the Ua metabolic profile, and these alterations did not appear to be related to oxidative stress.

## Data availability statement

The datasets presented in this study can be found in online repositories. The names of the repository/repositories and accession number(s) can be found below: OMIX004347 (OMIX, bioproject PRJCA017707; https://ngdc.cncb.ac.cn/omix/release/OMIX004347).

## Ethics statement

The study was approved by the human research ethics committees at the Sixth Medical Center (20/1/19) and Seventh Medical Center of the Chinese PLA General Hospital (20/1/19) and the PLA Strategic Support Force Characteristic Medical Center (18/2/20). The patients/participants provided their written informed consent to participate in this study.

## Author contributions

FC: conceptualization, methodology, investigation, writing-original draft, writing-review and editing, and visualization. TD and YL: data analysis, writing-original draft, and writing-review and editing. LZ: writing-original draft review and editing. LC and WJ: conceptualization, methodology, and writing-original draft. YZ and YC: conceptualization, methodology, investigation, writing-original draft, writing-review and editing, visualization, and funding acquisition. All authors contributed to the article and approved the submitted version.

## References

[1] Hamel MS Anderson BL Rouse DJ. Oxygen for intrauterine resuscitation: of unproved benefit and potentially harmful. Am J Obstet Gynecol (2014) 211(2):124–7. doi: 10.1016/j.ajog.2014.01.004 24412117

[2] Reddy UM Weiner SJ Saade GR Varner MW Blackwell SC Thorp JM Jr. Eunice Kennedy Shriver national institute of child health and human development (NICHD) maternal-fetal medicine units (MFMU) network. intrapartum resuscitation interventions for category II fetal heart rate tracings and improvement to category I. Obstet Gynecol (2021) 138(3):409–16. doi: 10.1097/AOG.0000000000004508 PMC8506980 34352857

[3] Burd JE Anderson K Berghella V Duncan DG Baxter JK Quist-Nelson J. Evaluation of an initiative to decrease the use of oxygen supplementation for category II fetal heart rate tracings. Obstet Gynecol (2021) 138(4):627–32. doi: 10.1097/AOG.0000000000004544 34623075

[4] Qian G Xu X Chen L Xia S Wang A Chuai Y. The effect of maternal low flow oxygen administration during the second stage of labour on umbilical cord artery pH: a randomised controlled trial. BJOG (2017) 124(4):678–85. doi: 10.1111/1471-0528.14418 28224745

[5] Thorp JA Trobough T Evans R Hedrick J Yeast JD. The effect of maternal oxygen administration during the second stage of labor on umbilical cord blood gas values: a randomized controlled prospective trial. Am J Obstet Gynecol (1995) 172:465–74. doi: 10.1016/0002-9378(95)90558-8 7856671

[6] Raghuraman N Wan L Temming LA Woolfolk C Macones GA Tuuli MG. Effect of oxygen vs room air on intrauterine fetal resuscitation: a randomized noninferiority clinical trial. JAMA Pediatr (2018) 172(9):818–23. doi: 10.1001/jamapediatrics.2018.1208 PMC6143068 30039159

[7] Moors S Bullens LM van Runnard Heimel PJ Dieleman JP Kulik W Bakkeren DL. The effect of intrauterine resuscitation by maternal hyperoxygenation on perinatal and maternal outcome: a randomized controlled trial. Am J Obstet Gynecol MFM (2020) 2(2):100102. doi: 10.1016/j.ajogmf.2020.100102 33345953

[8] Chuai Y Jiang W Zhang L Chuai F Sun X Peng K. Effect of long-duration oxygen vs room air during labor on umbilical cord venous partial pressure of oxygen: a randomized controlled trial. Am J Obstet Gynecol (2022) 227(4):629.e1–629.e16. doi: 10.1016/j.ajog.2022.05.028 35580635

[9] Sirimai K Atisook R Boriboonhirunsarn D. The correlation of intrapartum maternal oxygen administration and umbilical cord blood gas values. Acta Obstet Gynecol Scand Suppl (1997) 76(167:2):90.

[10] Chuai Y Jiang W Xu X Wang A Yao Y Chen L. Maternal oxygen exposure may not change umbilical cord venous partial pressure of oxygen: non-random, paired venous and arterial samples from a randomised controlled trial. BMC Pregnancy Childbirth (2020) 20(1):510. doi: 10.1186/s12884-020-03212-3 32887557 PMC7650259

[11] Watkins VY Martin S Macones GA Tuuli MG Cahill AG Raghuraman N. The duration of intrapartum supplemental oxygen administration and umbilical cord oxygen content. Am J Obstet Gynecol (2020) 223(3):440.e1–7. doi: 10.1016/j.ajog.2020.05.056 32497605

[12] Raghuraman N López JD Carter EB Stout MJ Macones GA Tuuli MG. The effect of intrapartum oxygen supplementation on category II fetal monitoring. Am J Obstet Gynecol (2020) 223(6):905.e1–7. doi: 10.1016/j.ajog.2020.06.037 32585226

[13] Allanson ER Waqar T White C Tunçalp Ö Dickinson JE. Umbilical lactate as a measure of acidosis and predictor of neonatal risk: a systematic review. BJOG (2017) 124(4):584–94. doi: 10.1111/1471-0528.14306 27704703

[14] Sanz-Cortés M Carbajo RJ Crispi F Figueras F Pineda-Lucena A Gratacós E. Metabolomic profile of umbilical cord blood plasma from early and late intrauterine growth restricted (IUGR) neonates with and without signs of brain vasodilation. PloS One (2013) 8(12):e80121. doi: 0.1371/journal.pone.0080121. eCollection 2013 24312458 10.1371/journal.pone.0080121 PMC3846503

[15] ACOG practice bulletin no. 106: intrapartum fetal heart rate monitoring: nomenclature, interpretation, and general management principles. Obstet Gynecol (2009) 114(1):192–202. doi: 10.1097/AOG.0b013e3181aef106 19546798

[16] ACOG committee on obstetric practice. ACOG committee opinion no. 348, November 2006: umbilical cord blood gas and acid-base analysis. Obstet Gynecol (2006) 108(5):1319–22. doi: 10.1097/00006250-200611000-00058 17077266

[17] Monneret D Desmurs L Zaepfel S Chardon L Doret-Dion M Cartier R. Reference percentiles for paired arterial and venous umbilical cord blood gases: an indirect nonparametric approach. Clin Biochem (2019) 67:40–7. doi: 10.1016/j.clinbiochem.2019.02.014 30831089

[18] Raghuraman N Temming LA Doering MM Stoll CR Palanisamy A Stout MJ. Maternal oxygen supplementation compared with room air for intrauterine resuscitation: a systematic review and meta-analysis. JAMA Pediatr (2021) 175(4):368–76. doi: 10.1001/jamapediatrics.2020.5351 PMC7783592 33394020

[19] Chatmongkolchart S Prathep S. Supplemental oxygen for caesarean section during regional anaesthesia. Cochrane Database Syst Rev (2016) 3:CD006161. doi: 10.1002/14651858.CD006161.pub3 26982519 PMC8735890

[20] Shao X Ye H Qiu X. Fifth edition: practice of neonatology. Beijing, China: People's Health Publishing House (2019).

[21] Polvi HJ Pirhonen JP Erkkola RU. The hemodynamic effects of maternal hypo- and hyperoxygenation in healthy term pregnancies. Obstet Gynecol (1995) 86(5):795–9. doi: 10.1016/0029-7844(95)00260-X 7566851

[22] James LS Morishima HO Daniel SS Bowe ET Cohen H Niemann WH. Mechanism of late deceleration of the fetal heart rate. Am J Obstet Gynecol (1972) 113(5):578–82. doi: 10.1016/0002-9378(72)90624-2 4631585

[23] Haydon ML Gorenberg DM Nageotte MP Ghamsary M Rumney PJ Patillo C. The effect of maternal oxygen administration on fetal pulse oximetry during labor in fetuses with nonreassuring fetal heart rate patterns. Am J Obstet Gynecol (2006) 195(3):735–8. doi: 10.1016/j.ajog.2006.06.084 16949405

[24] Wang C Gao Z. Internal medicine: respiratory and critical care medicine. Beijing, China: People's Health Publishing House (2016).

[25] Wang T. Ninth edition: physiology. Beijing, China: People's Health Publishing House (2018).

[26] Smit B Smulders YM van der Wouden JC Oudemans-van Straaten HM Spoelstra-de Man AME. Hemodynamic effects of acute hyperoxia: systematic review and meta-analysis. Crit Care (2018) 22(1):45. doi: 10.1186/s13054-018-1968-2 29477145 PMC6389225

[27] McHugh A El-Khuffash A Bussmann N Doherty A Franklin O Breathnach F. Hyperoxygenation in pregnancy exerts a more profound effect on cardiovascular hemodynamics than is observed in the nonpregnant state. Am J Obstet Gynecol (2019) 220(4):397.e1–8. doi: 10.1016/j.ajog.2019.02.059 30849354

[28] Higgins J Green S. Cochrane handbook for systematic reviews of interventions version 5.1.0. England: The Cochrane Collaboration (2011).

