# Supplementary material for: The effect of intrapartum prolonged oxygen exposure on fetal metabolic status: secondary analysis from a randomized controlled trial
Source: Front Endocrinol (Lausanne). 2023 Jun 27;14:1204956. doi: 10.3389/fendo.2023.1204956 (PMC10335765; doi:10.3389/fendo.2023.1204956)
Supplement: Supplementary file 1 [file DataSheet_1.doc]

Maternal Oxygen Administration for Fetal Distress II (Protocol)

ClinicalTrials.gov Identifier: NCT03764696

Recruitment Status: Completed

First Posted: December, 2018

Last Update Posted: December, 2021

Sponsor:

Sixth Medical Center (Former Navy General Hospital), Chinese PLA General Hospital , Beijing, China.

Principle Investigators:

Yunhai Chuai and Yuanqing Yao, Chinese PLA General Hospital, Medical School of Chinese PLA, Fuxing Road No. 28, Beijing 100853, China.

**Study Description**

Nowadays, maternal oxygen (O_2_) administration was the most common intervention for intrapartum resuscitation, it was estimated that more than half of women received O_2_ at some point during labor, although they were not hypoxic [1,2]. Obstetricians and midwives hoped that the additional O_2_ can improve the fetal acid-base metabolism status and relieve suspicious or abnormal fetal heart rate (FHR) tracings [1,2]. However, there was much controversy about the effects of therapeutic or prophylactic maternal O_2_ inhalation therapy during labor on neonatal outcomes [3,4]. Systematic reviews found low or very low-certainty evidence of hyperoxia-induced vasoconstriction in animal models and in healthy volunteers and patients [5,6]. The effects of hyperoxia on pregnant hemodynamics was studied in a cohort study, which found supplemental 80 to 100% fraction of inspired O_2_ (FiO_2_) for 10 minutes decreased cardiac index and increased systemic vascular resistance [7]. At present, there were only five randomized clinical trials (RCTs) of prophylactic or therapeutic O_2_ inhalation during labor showed that supplemental O_2_ did not improve fetal acid-base status or other neonatal outcomes, of these trials, women received prophylactic O_2_ during the second stage of labor (30 to 80% FiO_2_); or therapeutic O_2_ (80% FiO_2_) when they developed category II or III tracings at any point in the active phase or the second stage of labor until delivery [8-12].

An unproven hypothesis of O_2_ for intrauterine resuscitation is that the supra-physiological O_2_ can be transferred across the placenta to fetal circulation [13,14]. Fetal oxygenation was often tested by fetal pulse oximetry (FSpO_2_), blood O_2_ level-dependent magnetic resonance imaging (BOLD-MRI), and umbilical cord venous (Uv) partial pressure of O_2_ (PO_2_): for FSpO_2_, Dildy *et al.* [15] showed that supplemental 40% FiO_2_ (20 or 45 mins) resulted in no significant change in fetal O_2_ saturation, but other studies showed that 40 to 100% FiO_2_ (15 to 30 mins) increased fetal O_2_ saturation [16-18]; for BOLD-MRI, studies showed that a short time breathing 100% FiO_2_ (6 mins) increased cerebral blood oxygenation in fetuses with single ventricle or aortic obstruction, but 100% FiO_2_ did not affect fetal brain oxygenation (6 and 40 mins) or fetal liver oxygenation (15 mins) in healthy fetuses [19-21]; for Uv PO_2_, Thorp *et al.* [8] found no significant change in Uv PO_2_ with maternal 80% FiO_2_ for mean 45 minutes during the second stage of labor, but Watkins *et al.* [14] showed a significant decrease in Uv PO_2_ (median 25.5 versus 32.5 mmHg) with maternal 80% FiO_2_ over 176 minutes during the partial active phase and the second stage of labor.

In the real world, the duration of O_2_ exposure ranged from a few minutes to several hours, depending on clinical experience with no consensus [14]. We perform this trial to assess the effect of maternal long-duration high FiO_2_ exposure during labor on Uv PO_2_. The long-duration high FiO_2_ is defined as supplemental 60 to 80% FiO_2_ during the major part of labor (i.e. late latent phase, whole active phase and second stage of labor), and this duration is usually more than 3 hours.

To avoid pulmonary O_2_ toxicity, supplemental high FiO_2_ cannot exceed 24 hours [22], and the total duration of O_2_ exposure is not allowed to exceed 12 hours in our study.

**Study Design**

The study is a 3-site (based in Beijing, China), randomized, open-label, parallel-group trial of women in the latent phase of labor who had category I FHR tracings.

The study was approved by the human research ethics committees at Sixth Medical Center and Seventh Medical Center of Chinese PLA General Hospital, and PLA Strategic Support Force Characteristic Medical Center.

The trial was registered on ClinicalTrials.gov with the identifier NCT03764696 before its commencement.

Women will complete informed consent at the time of their presentation to the labor units.

| Study Type  : | Interventional  (Clinical Trial) |
| --- | --- |
| Estimated Enrollment  : | 140 participants |
| Allocation: | Randomized |
| Intervention Model: | Parallel Assignment |
| Masking: | None (Open Label) |
| Primary Purpose: | Prevention |
| Official Title: | The Effect of Maternal Long Term High Flow Oxygen Administration During Labor on Umbilical Cord Blood Gases |
| Actual Study Start Date : | January 1, 2021 |
| Estimated Primary Completion Date : | October 31, 2021 |
| Estimated Study Completion Date : | December 31, 2021 |

Contacts and Locations

Contact: Yunhai Chuai, Dr +86-18810892004 wangyh85@foxmail.com

Locations

| Department of Obstetrics and Gynecology, Sixth Medical Center, Chinese PLA General Hospital, Beijing, China  Contact: Yunhai Chuai  Principal Investigator: Yunhai Chuai, Dr  Sub-Investigator: Lei Chen, Dr |
| --- |
| Department of Obstetrics and Gynecology, Seventh Medical Center, Chinese PLA General Hospital, Beijing, China  Contact: Wen Jiang  Sub-Investigator: Wen Jiang  Sub-Investigator: Yuanqing Yao |
| Department of Obstetrics and Gynecology, PLA Strategic Support Force Characteristic Medical Center, Beijing, China  Contact: Lanmei Zhang  Sub-Investigator: Lanmei Zhang  Sub-Investigator: Kefei Peng |

**Eligibility Criteria**

| Ages Eligible for Study: | 18 Years to 40 Years   (Adult) |
| --- | --- |
| Sexes Eligible for Study: | Female |
| Accepts Healthy Volunteers: | Yes |

Criteria

Inclusion Criteria:

at term (>37 weeks, <42 weeks), singleton, cephalic presentation, spontaneous or induced labor, normal labor, category I FHR tracings, 2 to 3 cm of cervical dilation in nulliparity, 1 to 2 cm of cervical dilation in multipara, informed consent.

Exclusion Criteria:

respiratory or cardiovascular disease, diabetes mellitus or insulin-treated gestational diabetes mellitus, hypertension or preeclampsia, oligohydramnios, fetal growth restriction, placental abruption, cephalopelvic disproportion, meconium-stained amniotic fluid, tachysystole, having received O_2_, uterine incision (myomectomy or perforation), anemia, fever, chorioamnionitis, tobacco or alcohol use, disorders in oxygen saturations, hypotension, uncomfortable with facemask.

**Randomization**

Obstetricians assess women for eligibility. Consented women are randomized at 2 to 3 cm of cervical dilation in nulliparity and 1 to 2 cm of cervical dilation in multipara in the latent phase of labor. Participants are randomly assigned to a computer-generated random sequence that use balanced variable blocks within each site in a ratio of 1:1. Random sequence is generated by one investigator at each site who take no further part in this study. Randomization is performed by the sequential opening of sealed numbered envelopes by one research nurse at each site.

**Arms and Interventions**

| Arm | Intervention/treatment |
| --- | --- |
| No Intervention: air, the first and second stage of labor  Patients randomized to the group will receive room air.  The therapy will continue until after delivery |  |
| Experimental: oxygen, the first and second stage of labor  Patients randomized to the group will receive oxygen administered by high flow facemask oxygen at 10 L/min oxygen.  The therapy will continue until after delivery | Device: tight-fitting simple facemask  Oxygen will be administered by facemask at 10 L/min oxygen. The therapy will continue until after delivery |

Women are assigned to receive either O_2_ via the tight-fitting simple facemask at a flow rate of 10 L per minute (FiO_2_ was about 60 to 80% [8,11,12]) until delivery, or room air only without a facemask. The facemask is administered by one research nurse to ensure that it is worn properly to cover the nose and mouth. Supplemental O_2_ can be interrupted for some reasons such as eating, drinking, and etc., but the total duration cannot exceed 15 minutes. To avoid O_2_ toxicity, the total duration of O_2_ exposure is not allowed to exceed 12 hours, otherwise the intervention will be terminated. All participants will receive standard intrapartum care and give birth in a supine position. Electronic fetal monitoring is tested every 15 to 30 minutes in the first stage of labor and continuously during the second stage. A three-tiered interpretation recommended by the American College of Obstetricians and Gynecologists (ACOG) is used to manage category II or III FHR tracings [23], and the techniques of intrauterine resuscitation for both groups including changing maternal position, discontinuation of oxytocin, intravenous fluid bolus, and tocolytic agents. Amnioinfusion will not be used in this study.

**Data Collection**

Baseline characteristics of study participants are collected, including maternal age, gestational age, nulliparity or multipara, body mass index (BMI), antepartum hematocrit, spontaneous or induced labor, pain management, augmentation of labor.

**Outcome Measures**

Primary Outcome Measures:

The primary outcome is the Uv PO_2_. The paired Uv and umbilical cord arterial (Ua) blood samples will be collected as recommended by ACOG [24]. A segment of umbilical cord (20 cm) is double-clamped, divided, and blood samples were obtained by trained technicians immediately after delivery, and these samples are analyzed in the delivery units using the Gem Premier 4000 benchtop blood gas analyzer (Werfen America).

We validate the umbilical cord blood gases according to the criteria of Monneret et al. [25], who established three exclusion criteria: an Uv - Ua pH difference < 0.02, an Ua - Uv partial pressure of carbon dioxide (PCO_2_) < 5.25 mmHg, and an Uv PCO_2_ < 21.75 mmHg.

| Cord arterial pH values [ Time Frame: within 30 to 60 seconds of birth ]  Immediately after delivery (within 30-60 seconds of birth), blood gas sample will be obtained. |
| --- |
| Cord arterial partial pressure of oxygen [ Time Frame: within 30 to 60 seconds of birth ]  Immediately after delivery (within 30-60 seconds of birth), blood gas sample will be obtained. |
| Cord arterial partial pressure of carbon dioxide [ Time Frame: within 30 to 60 seconds of birth ]  Immediately after delivery (within 30-60 seconds of birth), blood gas sample will be obtained. |
| Cord venous pH values [ Time Frame: within 30 to 60 seconds of birth ]  Immediately after delivery (within 30-60 seconds of birth), blood gas sample will be obtained. |
| Cord venous partial pressure of oxygen [ Time Frame: within 30 to 60 seconds of birth ]  Immediately after delivery (within 30-60 seconds of birth), blood gas sample will be obtained. |
| Cord venous partial pressure of carbon dioxide [ Time Frame: within 30 to 60 seconds of birth ]  Immediately after delivery (within 30-60 seconds of birth), blood gas sample will be obtained. |

Secondary Outcome Measures:

| Rate of abnormal fetal heart tracing [ Time Frame: at 1 minute after birth ]  Secondary outcomes are women who develop category II or III FHR tracings in labor. Two trained research nurses, unaware of randomization assignments, assess FHR tracings in the storage computer independently, and resolve disagreements by discussion with a third nurse. Details of II or III FHR tracings are recorded, including baseline, variability, and deceleration.  Three-Tiered Fetal Heart Rate Interpretation System  Category I  • Category I FHR tracings include all of the following:  • Baseline rate: 110–160 beats per minute  • Baseline FHR variability: moderate  • Late or variable decelerations: absent  • Early decelerations: present or absent  • Accelerations: present or absent  Category II  Category II FHR tracings includes all FHR tracings not categorized as Category I or Category III. Category II tracings may represent an appreciable fraction of those encountered in clinical care.  Examples of Category II FHR tracings include any of the following:  Baseline rate  • Bradycardia not accompanied by absent baseline variability  • Tachycardia  Baseline FHR variability  • Minimal baseline variability  • Absent baseline variability with no recurrent decelerations  • Marked baseline variability  Accelerations  • Absence of induced accelerations after fetal stimulation  Periodic or episodic decelerations  • Recurrent variable decelerations accompanied by minimal or moderate baseline variability  • Prolonged deceleration more than 2 minutes but less than10 minutes  • Recurrent late decelerations with moderate baseline variability  • Variable decelerations with other characteristics such as slow return to baseline, overshoots, or “shoulders”  Category III  Category III FHR tracings include either  • Absent baseline FHR variability and any of the following:  —Recurrent late decelerations  —Recurrent variable decelerations  —Bradycardia  • Sinusoidal pattern |
| --- |

Other Outcome Measures:

| Rate of cesarean delivery [ Time Frame: at 1 minute after birth ] |
| --- |
| Rate of assisted vaginal delivery [ Time Frame: at 1 minute after birth ] |
| Apgar score less than 7 [ Time Frame: at 1 and 5 minutes after birth ]  The Apgar scale is determined by evaluating the newborn baby on five simple criteria on a scale from 0 to 2, then summing up the five values thus obtained. The resulting Apgar score ranges from 0 to 10. The five criteria are summarized using words chosen (Appearance, Pulse, Grimace, Activity, Respiration). The infant is given a score of 0, 1 or 2. The scores are added up and the total sum is their Apgar score.  The test is generally done at one and five minutes after birth, and may be repeated later if the score is and remains low. Scores 7 and above are generally normal, 4 to 6 fairly low, and 3 and below are generally regarded as critically low. |
| Rate of serious neonatal morbidity or death [ Time Frame: within 28 days of birth ] |
| Cord arterial plasma liquid chromatography mass spectrometry analysis [ Time Frame: within 30 to 60 seconds of birth ]  The systematic identification and quantitation of all the metabolic products (mainly endogenous small molecule compounds with relative molecular weight within 1000 Da) of cord arterial plasma under oxygen and room air conditions. Immediately after delivery (within 30-60 seconds of birth), blood plasma sample will be obtained and the analysis will be performed by the Novogene Co., Ltd. |

**Sample size**

Sample size is calculated based on the Uv PO_2_. According to our institutional data and literature, we estimate a baseline Uv PO_2_ mean ± standard deviation of 27 ± 6 mmHg in term infants without pathological pregnancies or complicated deliveries during labor [25]. The Cochrane review (including 10 RCTs, 683 participants) showed the short-duration supplementary O_2_ could significantly increase Uv PO_2_ (FiO_2_ at least 60%, MD 8.19 mmHg, and FiO_2_ less than 60%, MD 3.95 mmHg) during caesarean section, however, it was a very low-certainty evidence due to small sample studies and high heterogeneity [26]. The findings from two RCTs (2 RCTs, 200 participants) reported the short-duration 80% FiO_2_ did not affect Uv/Ua PO_2_ during labor [8,14], and the secondary analysis of one trial (12 non-random samples) found that prolonged O_2_ exposure (over 176 mins) was associated with lower Uv PO_2_ (median 25.5 versus 32.5 mmHg) [14]. In the present trial, a clinically significant change of Uv PO_2_ is assumed to be 3 mmHg units. To detect the 3 mmHg difference in Uv PO_2_ with 80% power and a 2-sided test of 0.05, we estimate that 64 women were needed in each group. We anticipate a 10% loss to unattainable paired cord gases, resulting in our plan to enroll 140 participants.

**Statistical Analysis**

Data analysis for the primary outcomes will be performed using the modified intention-to-treat principle, participants with unvalidate paired cord blood gases will be excluded. The paired cord blood gases samples will be collected as recommended by ACOG [24]. The blood samples are obtained by trained technicians, and the rate of loss to follow-up is expected to be is less than 5%. According to our experience, of those who will be enrolled, the cesarean delivery rate is less than 5%, and their duration of labor will be recorded based on the results of the last observation.

Per protocol analyses will be performed if randomized women receive their assigned interventions with interruption or crossover.

The distribution of continuous variables will be analyzed using the Kolmogorov-Smirnov test.

Student’s *t* test or Mann-Whitney *U* test will be used as appropriate.

Categorical variables will be analyzed using Chi-square test or Fisher exact test.

Data will be expressed as mean (standard deviation), median (IQR), or number (%).

All statistical analyses will be performed using SPSS version 19 (IBM SPSS Statistics).

**References**

1. Hamel MS, Anderson BL, Rouse DJ. Oxygen for intrauterine resuscitation: of unproved benefit and potentially harmful. Am J Obstet Gynecol. 2014 Aug;211(2):124-7.
2. Reddy UM, Weiner SJ, Saade GR, Varner MW, Blackwell SC, Thorp JM Jr, Tita ATN, Miller RS, Peaceman AM, McKenna DS, Chien EKS, Rouse DJ, El-Sayed YY, Sorokin Y, Caritis SN; Eunice Kennedy Shriver National Institute of Child Health and Human Development (NICHD) Maternal-Fetal Medicine Units (MFMU) Network. Intrapartum Resuscitation Interventions for Category II Fetal Heart Rate Tracings and Improvement to Category I. Obstet Gynecol. 2021 Sep 1;138(3):409-416.
3. Fawole B, Hofmeyr GJ. Maternal oxygen administration for fetal distress. Cochrane Database Syst Rev. 2012 Dec 12;12(12):CD000136.
4. Raghuraman N, Temming LA, Doering MM, Stoll CR, Palanisamy A, Stout MJ, Colditz GA, Cahill AG, Tuuli MG. Maternal Oxygen Supplementation Compared With Room Air for Intrauterine Resuscitation: A Systematic Review and Meta-analysis. JAMA Pediatr. 2021 Apr 1;175(4):368-376.
5. Smit B, Smulders YM, Eringa EC, Oudemans-van Straaten HM, Girbes ARJ, Wever KE, Hooijmans CR, Spoelstra-de Man AME. Effects of hyperoxia on vascular tone in animal models: systematic review and meta-analysis. Crit Care. 2018 Aug 4;22(1):189.
6. Smit B, Smulders YM, van der Wouden JC, Oudemans-van Straaten HM, Spoelstra-de Man AME. Hemodynamic effects of acute hyperoxia: systematic review and meta-analysis. Crit Care. 2018 Feb 25;22(1):45.
7. McHugh A, El-Khuffash A, Bussmann N, Doherty A, Franklin O, Breathnach F. Hyperoxygenation in pregnancy exerts a more profound effect on cardiovascular hemodynamics than is observed in the nonpregnant state. Am J Obstet Gynecol. 2019 Apr;220(4):397.e1-397.e8.
8. Thorp JA, Trobough T, Evans R, Hedrick J, Yeast JD. The effect of maternal oxygen administration during the second stage of labor on umbilical cord blood gas values: a randomized controlled prospective trial. Am J Obstet Gynecol. 1995;172:465-74.
9. Sirimai K, Atisook R, Boriboonhirunsarn D. The correlation of intrapartum maternal oxygen administration and umbilical cord blood gas values. Acta Obstetricia et Gynecologica Scandinavica Supplement. 1997;76(167:2):90.
10. Qian G, Xu X, Chen L, Xia S, Wang A, Chuai Y, Jiang W. The effect of maternal low flow oxygen administration during the second stage of labour on umbilical cord artery pH: a randomised controlled trial. BJOG. 2017 Mar;124(4):678-685.
11. Raghuraman N, Wan L, Temming LA, Woolfolk C, Macones GA, Tuuli MG, Cahill AG. Effect of Oxygen vs Room Air on Intrauterine Fetal Resuscitation: A Randomized Noninferiority Clinical Trial. JAMA Pediatr. 2018 Sep 1;172(9):818-823.
12. Moors S, Bullens LM, van Runnard Heimel PJ, Dieleman JP, Kulik W, Bakkeren DL, van den Heuvel ER, van der Hout-van der Jagt MB, Oei SG. The effect of intrauterine resuscitation by maternal hyperoxygenation on perinatal and maternal outcome: a randomized controlled trial. Am J Obstet Gynecol MFM. 2020 May;2(2):100102.
13. Raghuraman N, López JD, Carter EB, Stout MJ, Macones GA, Tuuli MG, Cahill AG. The effect of intrapartum oxygen supplementation on category II fetal monitoring. Am J Obstet Gynecol. 2020 Dec;223(6):905.e1-905.e7.
14. Watkins VY, Martin S, Macones GA, Tuuli MG, Cahill AG, Raghuraman N. The duration of intrapartum supplemental oxygen administration and umbilical cord oxygen content. Am J Obstet Gynecol. 2020 Sep;223(3):440.e1-440.e7.
15. Dildy GA, Clark SL, Loucks CA. Intrapartum fetal pulse oximetry: the effects of maternal hyperoxia on fetal arterial oxygen saturation. Am J Obstet Gynecol. 1994 Oct;171(4):1120-4
16. Simpson KR, James DC. Efficacy of intrauterine resuscitation techniques in improving fetal oxygen status during labor. Obstet Gynecol. 2005 Jun;105(6):1362-8.
17. Haydon ML, Gorenberg DM, Nageotte MP, Ghamsary M, Rumney PJ, Patillo C, Garite TJ. The effect of maternal oxygen administration on fetal pulse oximetry during labor in fetuses with nonreassuring fetal heart rate patterns. Am J Obstet Gynecol. 2006 Sep;195(3):735-8.
18. Aldrich CJ, Wyatt JS, Spencer JA, Reynolds EO, Delpy DT. The effect of maternal oxygen administration on human fetal cerebral oxygenation measured during labour by near infrared spectroscopy. Br J Obstet Gynaecol. 1994 Jun;101(6):509-13.
19. Huen I, Morris DM, Wright C, Sibley CP, Naish JH, Johnstone ED. Absence of PO2 change in fetal brain despite PO2 increase in placenta in response to maternal oxygen challenge. BJOG. 2014 Dec;121(13):1588-94.
20. You W, Andescavage NN, Kapse K, Donofrio MT, Jacobs M, Limperopoulos C. Hemodynamic Responses of the Placenta and Brain to Maternal Hyperoxia in Fetuses with Congenital Heart Disease by Using Blood Oxygen-Level Dependent MRI. Radiology. 2020 Jan;294(1):141-148.
21. Morris DM, Ross JA, McVicar A, Semple SI, Haggarty P, Gilbert FJ, Abramovich DR, Smith N, Redpath TW. Changes in foetal liver T2* measurements by MRI in response to maternal oxygen breathing: application to diagnosing foetal growth restriction. Physiol Meas. 2010 Sep;31(9):1137-46.
22. Wang C, Gao Z. Internal medicine: respiratory and critical care medicine. Beijing, China: People's Health Publishing House. 2016.
23. ACOG Practice Bulletin No. 106: Intrapartum fetal heart rate monitoring: nomenclature, interpretation, and general management principles. Obstet Gynecol. 2009 Jul;114(1):192-202.
24. ACOG Committee on Obstetric Practice. ACOG Committee Opinion No. 348, November 2006: Umbilical cord blood gas and acid-base analysis. Obstet Gynecol. 2006 Nov;108(5):1319-22.
25. Monneret D, Desmurs L, Zaepfel S, Chardon L, Doret-Dion M, Cartier R. Reference percentiles for paired arterial and venous umbilical cord blood gases: An indirect nonparametric approach. Clin Biochem. 2019 May;67:40-47.
26. Chatmongkolchart S, Prathep S. Supplemental oxygen for caesarean section during regional anaesthesia. Cochrane Database Syst Rev. 2016 Mar 16;3:CD006161.
