# Supplementary material for: The effect of intrapartum prolonged oxygen exposure on fetal metabolic status: secondary analysis from a randomized controlled trial
Source: Front Endocrinol (Lausanne). 2023 Jun 27;14:1204956. doi: 10.3389/fendo.2023.1204956 (PMC10335765; doi:10.3389/fendo.2023.1204956)
Supplement: Supplementary file 2 [file DataSheet_2.pdf]

Untargeted Metabolomics - Materials and Methods

1. Metabolites Extraction

Tissue sample

Tissues (100 mg) were individually grounded with liquid nitrogen and the homogenate was resuspended with prechilled 80% methanol by well vortex. The sampleswere incubated on ice for 5 min and then were centrifuged at 15,000 g, 4°C for 20 min. Some of supernatant was diluted to final concentration containing 53% methanol by LC-MS grade water.The samples were subsequently transferred to a fresh Eppendorf tube and thenwere centrifuged at 15000 g, 4°C for 20 min. Finally, the supernatant was injected into the LC-MS/MS system analysis[1].

Liquid sample

The samples (100 μL) were placed in the EP tubes and resuspended with prechilled 80% methanol by well vortex. Then the samples were incubated on ice for 5 min and centrifuged at 15,000 g, 4°C for 20 min. Some of supernatant was diluted to final concentration containing 53% methanol by LC-MS grade water.The samples were subsequently transferred to a fresh Eppendorf tube and then were centrifuged at 15000 g, 4°C for 20 min. Finally, the supernatant was injected into the LC-MS/MS system analysis[2-3].

Cell or bacteria sample

The samples were placed in the EP tubes and resuspended with prechilled 80% methanol by well vortex. Then the samples were melted on ice and whirled for 30 s. After the sonification for 6 min, they were centrifuged at 5,000 rpm, 4°C for 1 min. The supernatant was freeze-dried and dissolvedwith 10% methanol. Finally, the solution was injected into the LC-MS/MS system analysis[4-5].

Cell or bacteria culture medium sample

The samples (1 mL) were freeze-dried and resuspended with prechilled 80% methanol by well vortex. Then the samples were incubated on ice for 5 min and centrifuged at 15,000 g, 4°C for 15 min. Some of supernatant was diluted to final concentration containing 53% methanol by LC-MS grade water.The samples were subsequently

transferred to a fresh Eppendorf tube and then were centrifuged at 15000 g, 4°C for 15 min. Finally, the supernatant was injected into the LC-MS/MS system analysis.

2. UHPLC-MS/MS Analysis

UHPLC-MS/MS analyses were performed using a Vanquish UHPLC system (ThermoFisher, Germany) coupled with an Orbitrap Q Exactive^{TM}HF-X mass spectrometer (Thermo Fisher，Germany) in Novogene Co., Ltd. (Beijing, China). Samples were injected onto a Hypesil Gold column (100×2.1 mm, 1.9μm) using a 17-min linear gradient at a flow rate of 0.2mL/min. The eluents for the positive polarity mode were eluent A (0.1% FA in Water) and eluent B (Methanol).The eluents for the negative polarity mode were eluent A (5 mMammonium acetate, pH 9.0) and eluent B (Methanol).The solvent gradient was set as follows: 2% B, 1.5 min; 2-85% B, 3 min; 100% B, 10 min；100-2% B, 10.1 min；2% B, 12 min. Q Exactive^{TM} HF mass spectrometer was operated in positive/negative polarity mode with spray voltage of 3.2 kV, capillary temperature of 320°C, sheath gas flow rate of 40 arb and aux gas flow rate of 10 arb, Funnel RF level of 40, Aux gas heater temperature of 350°C.

3. Data processing and metabolite identification

The raw data files generated by UHPLC-MS/MS were processed using the Compound Discoverer 3.1 (CD3.1, ThermoFisher) to perform peak alignment, peak picking, and quantitation for each metabolite. The main parameterswere set as follows: retention time tolerance, 0.2 minutes; actual mass tolerance, 5ppm; signal intensity tolerance, 30%; signal/noise ratio, 3; and minimum intensity, et al. After that, peak intensities were normalized to the total spectral intensity.The normalized data was used to predict the molecular formula based on additive ions, molecular ion peaks and fragment ions. And then peaks were matched with the mzCloud (https://www.mzcloud.org/)，mzVault and MassList database to obtain the accurate qualitative and relative quantitative results.Statistical analyses were performed using the statistical software R (R version R-3.4.3),Python (Python 2.7.6 version) and CentOS (CentOS release 6.6),When data were not normally distributed, normal transformations were attempted using of area normalization method.

4. Data Analysis

These metabolites were annotated using the KEGG database (https://www.genome.jp/kegg/pathway.html), HMDB database (https://hmdb.ca/metabolites) and LIPIDMaps database (http://www.lipidmaps.org/). Principal components analysis (PCA) and Partial least squares discriminant analysis (PLS-DA) were performed at metaX[6] (a flexible and comprehensive software for processing metabolomics data).We applied univariate analysis (t-test) to calculate the statistical significance (P-value).The metabolites with VIP > 1 and P-value< 0.05 and fold change≥2 or FC≤0.5 were considered to be differential metabolites. Volcano plots were used to filter metabolites of interest which based on log_2(FoldChange) and -log_{10}(p-value) of metabolites by ggplot2 in R language.

For clustering heat maps, the data were normalized using z-scores of the intensity areas of differential metabolites and were ploted by Pheatmap package in R language. The correlation between differential metabolites were analyzed by cor () in R language (method=pearson).Statistically significant of correlation between differential metabolites were calculated by cor.mtest() in R language. P-value < 0.05 was considered as statistically significant and correlation plots were ploted by corrplot package in R language.The functions of these metabolites and metabolic pathways were studied using the KEGG database.The metabolic pathways enrichment of differential metabolites was performed, when ratio were satisfied by x/n > y/N, metabolic pathway were considered asenrichment, when P-value of metabolic pathway < 0.05, metabolic pathway were considered as statistically significant enrichment.

Reference

[1] Want E J , Masson P , Michopoulos F , et al. Global metabolic profiling of animal and human tissues via UPLC-MS[J]. Nature Protocols, 2012, 8(1):17-32.

[2] Want E J , O"Maille G , Smith C A , et al. Solvent-Dependent Metabolite Distribution, Clustering, and Protein Extraction for Serum Profiling with Mass Spectrometry[J]. Analytical Chemistry, 2006, 78(3):743-752.

[3] BarriT ,Dragsted L O . UPLC-ESI-QTOF/MS and multivariate data analysis for blood plasma

and serum metabolomics: effect of experimental artefacts and anticoagulant[J]. Analytica Chimica Acta, 2013, 768(1):118-128.
[4] Sellick C A, Hansen R, Stephens G M, et al. Metabolite extraction from suspension cultured mammalian cells for global metabolite profiling[J]. Nature Protocol, 2011, 6(8):1241-9.
[5] Yuan M, Breitkopf S B, Yang X, et al. A positive/negative ion-switching, targeted mass spectrometry-based metabolomics platform for bodily fluids, cells, and fresh and fixed tissue[J]. Nature Protocols, 2012, 7(5):872-81.
[6] Wen B , Mei Z , Zeng C , et al. metaX: a flexible and comprehensive software for processing metabolomics data[J]. BMC Bioinformatics, 2017, 18(1).
