# Supplementary material for: The effect of intrapartum prolonged oxygen exposure on fetal metabolic status: secondary analysis from a randomized controlled trial
Source: Front Endocrinol (Lausanne). 2023 Jun 27;14:1204956. doi: 10.3389/fendo.2023.1204956 (PMC10335765; doi:10.3389/fendo.2023.1204956)
Supplement: Supplementary file 3 [file DataSheet_3.zip › Result-X101SC21092977-Z01-J001-B1-42/Result_list-Software_readme.pdf]

Result_list-Software_readme

1. **结果目录结构：**

|-- 1.MetQuant-QC 【代谢物定性定量结果目录】

| |-- meta_intensity_{pos,neg,all}.xls 【所有代谢物定量结果列表】
| |-- sam_infor_{pos,neg,all}.xls 【样本信息及总 PCA 图中样本编号信息】
| |-- Samples_QC_{pos,neg,all}-PCA[.3D].{png,pdf} 【QC 及所有代谢样本的总 PCA 图】
| |-- Samples_ QC _pcaloading_{pos,neg,all}.{png,pdf}
| |--【QC 及所有代谢样本总 PCA 图的载荷图】
| |-- Samples_{pos,neg,all}-PCA[.3D].{png,pdf} 【所有代谢样本的总 PCA 图】
| |-- Samples_pcaloading_{pos,neg,all}.{png,pdf} 【所有代谢样本总 PCA 图的载荷图】
| |-- **【TIC】**
| |-- Samples_Specialized_Traces_{pos,neg,all}.xls【QC 及样本的 TIC 图】
| |-- **【Correlation】**
| |-- cor_pearson_{pos,neg,all}.{png,pdf,xls} 【QC 样本相关性分析结果】

|-- 2. MetAnnotation 【代谢物注释结果目录】

| -- KEGG 【基于 KEGG 数据库注释代谢物通路结果】

| |--meta_{pos,neg,all}_kegg_anno_category.xls 【KEGG 通路注释结果】
| |--meta_{pos,neg,all}.KEGG.Anno. {png,pdf} 【KEGG 注释结果统计图】
| |---meta_{pos,neg,all}.KEGG.Anno.xls 【代谢物 KEGG 注释结果列表】
| -- HMDB 【基于 HMDB 数据库注释代谢物分类结果】
| |--meta_{pos,neg,all}_hmdb_anno_category.xls【HMDB 分类注释结果】
| |--meta_{pos,neg,all}.HMDB.Anno.{png,pdf} 【HMDB 分类注释结果统计图】
| |-- meta_{pos,neg,all}.HMDB.Anno.xls 【代谢物 HMDB 注释结果列表】
| -- Lipidmaps 【基于 Lipidmaps 数据库注释代谢物分类结果】
| |--meta_{pos,neg,all}_lipidmaps_anno_category.xls【Lipidmaps 分类注释结果】
| |--meta_{pos,neg,all}.Lipidmaps.Anno.{png,pdf} 【Lipidmaps 分类注释结果统计图】
| |-- meta_{pos,neg,all}.Lipidmaps.Anno.xls 【代谢物 Lipidmaps 注释结果列表】| -- HMDB_KEGG_Lipidmaps 【基于 KEGG、HMDB、Lipidmaps 数据库注释整合结果】
| -- meta_intensity _{pos,neg,all}_hmdb_kegg_lipidmaps.xls【总 HMDB、KEGG、Lipimaps 数据库注释整合结果】

|-- 3.MetDiffScreening 【差异代谢物筛选结果目录】

| -- *.vs.* 【样本比较对目录】
| |-- *.vs.*_{pos,neg,all}.xls 【样本比较对定量分析结果】
| |-- *.vs.*_{pos,neg,all}_Diff.xls 【样本比较对差异代谢物分析】
| |-- *.vs.*_{pos,neg,all}_diff.anno.xls 【样本比较对差异代谢物注释结果】
| |-- *.vs.*_{pos,neg,all}-PCA[.3D].{png,pdf} 【样本比较对 PCA 分析】
| |-- *.vs.*_{pos,neg,all}-PCA-pcaloading.{png,pdf} 【样本比较对 PCA loading 分析】

| |-- *.vs.*_{pos,neg,all}-PLSDA-{score,valid}.{png,pdf}【比较对 PLSDA 分析】
| |-- *.vs.*_{pos,neg,all}-PLSDA-loading.{png,pdf}【比较对 PLSDA loading 分析】
| |-- *.vs.*_{pos,neg,all}.xls.volcano.{png,pdf} 【样本比较对火山图分析】

|-- 4.MetDiffAnalysis 【差异代谢物分析结果目录】

| -- Heatmap_diff 【总差异代谢物热图结果】
| |--Diff_Heatmap_{pos,neg,all}{_cluster,cluster_detail}.{png,pdf}【总差异代谢物聚类热图】
| |--Diff_Heatmap_{pos,neg,all}{,_detail}.{png,pdf}【总差异代谢物不聚类热图】| -- *.vs.* 【比较的样本对目录】
| |-- *.vs.*_{pos,neg,all}.corr.xls 【差异代谢物相关性列表】
| |-- *.vs.*_{pos,neg,all}_Pvalue.xls 【基于相关性分析的 p 值列表】
| |-- *.vs.*_{pos,neg,all}_zscore.xls 【差异代谢物 zscore 分析列表】
| |-- *.vs.*_{pos,neg,all}_zscore.{png,pdf} 【差异代谢物 z-score 图】
| |-- | |*.vs.*_{pos,neg,all}_cluster_heatmap[,_detail].{png,pdf} 【差异代谢物聚类热图】
| |-- *.vs.*_{pos,neg,all}_heatmap[._detail].{png,pdf} 【差异代谢物不聚类热图】
| |-- ROC_{pos,neg,all} 【差异代谢物 ROC 曲线图】

|-- 5.MetKeggEnrichment 【差异代谢物 KEGG 富集分析结果目录】

| -- *.vs.* 【比较的样品对目录】
| |-- *.vs.*_{pos,neg,all}.kegg_enrichment.xls【差异代谢物 kegg 富集结果列表】
| |-- *.vs.*_{pos,neg,all}.KEGG_Enrich.scatterplot.{png,pdf}【kegg 富集气泡图】
| |-- *.vs.*_{pos,neg,all}_KEGG_map 【kegg 通路图】
|--Met_results_dir.html 【网页版结果文件链接】

2. 分析过程使用的软件版本汇总

<table>
<tr><th>分析</th><th>内容</th><th>软件</th><th>版本</th></tr>
<tr><td rowspan="2">代谢物定性定量</td><td>代谢物定性</td><td rowspan="2">Python</td><td rowspan="2">Python-3.5.0</td></tr>
<tr><td>代谢物定量</td></tr>
<tr><td rowspan="2">数据质控</td><td>QC样本质控</td><td rowspan="2">R</td><td rowspan="2">R-3.4.3</td></tr>
<tr><td>总样品PCA分析</td></tr>
<tr><td rowspan="2">代谢物注释</td><td rowspan="2">KEGG\HMDB\LIPIDMAPS</td><td>python</td><td>Python-2.7.6</td></tr>
<tr><td>R</td><td>R-3.4.3</td></tr>
<tr><td rowspan="3">差异代谢物筛选</td><td>主成分分析（PCA）</td><td rowspan="6">python、R</td><td rowspan="6">Python-3.5.0、R-3.4.3</td></tr>
<tr><td>偏最小二乘法判别分析（PLS-DA）</td></tr>
<tr><td>火山图</td></tr>
<tr><td rowspan="3">差异代谢物分析</td><td>差异代谢物聚类分析</td></tr>
<tr><td>差异代谢物相关性分析</td></tr>
<tr><td>Z-score分析</td></tr>
<tr><td rowspan="2">KEGG富集分析</td><td rowspan="2">KEGG富集</td><td>python</td><td>Python-3.5.0</td></tr>
<tr><td>R</td><td>R-3.4.3</td></tr>
<tr><td>差异代谢物ROC曲线分析</td><td>ROC曲线</td><td>R</td><td>R-3.4.3</td></tr>
<tr><td rowspan="4">关联分析</td><td rowspan="2">相关性分析</td><td>python</td><td>Python-3.5.0</td></tr>
<tr><td>R</td><td>R-3.4.3</td></tr>
<tr><td rowspan="2">KEGG共有通路筛选</td><td>python</td><td>Python-3.5.0</td></tr>
<tr><td>R</td><td>R-3.4.3</td></tr>
<tr><td>备注</td><td colspan="3">所有分析内容，数据处理主要用Python，图表绘制用R语言</td></tr>
</table>
