# Supplementary material for: The effect of intrapartum prolonged oxygen exposure on fetal metabolic status: secondary analysis from a randomized controlled trial
Source: Front Endocrinol (Lausanne). 2023 Jun 27;14:1204956. doi: 10.3389/fendo.2023.1204956 (PMC10335765; doi:10.3389/fendo.2023.1204956)
Supplement: Supplementary file 3 [file DataSheet_3.zip › Result-X101SC21092977-Z01-J001-B1-42/5.MetKeggEnrichment/O.vs.A/O.vs.A_neg_KEGG_map/KEGG.map.html]

Pathway Enrichment

  

# The most enriched pathway terms of O.vs.A\_neg

Statistic method: hypergeometric test

FDR correction method: Benjamini and Hochberg

| MapID | MapTitle | Pvalue | AdjustedPv | x | y | n | N | EnrichDirect | GeneIDs |
| --- | --- | --- | --- | --- | --- | --- | --- | --- | --- |
| map00380 | Tryptophan metabolism | 0.0459770114942529 | 0.0715197956577267 | 1 | 1 | 4 | 87 | Over | Com\_6399\_neg |
| map00740 | Riboflavin metabolism | 0.0459770114942529 | 0.0715197956577267 | 1 | 1 | 4 | 87 | Over | Com\_5716\_neg |
| map00790 | Folate biosynthesis | 0.0459770114942529 | 0.0715197956577267 | 1 | 1 | 4 | 87 | Over | Com\_5716\_neg |
| map03013 | RNA transport | 0.0459770114942529 | 0.0715197956577267 | 1 | 1 | 4 | 87 | Over | Com\_5716\_neg |
| map04014 | Ras signaling pathway | 0.0459770114942529 | 0.0715197956577267 | 1 | 1 | 4 | 87 | Over | Com\_5716\_neg |
| map04015 | Rap1 signaling pathway | 0.0459770114942529 | 0.0715197956577267 | 1 | 1 | 4 | 87 | Over | Com\_5716\_neg |
| map04122 | Sulfur relay system | 0.0459770114942529 | 0.0715197956577267 | 1 | 1 | 4 | 87 | Over | Com\_5716\_neg |
| map04144 | Endocytosis | 0.0459770114942529 | 0.0715197956577267 | 1 | 1 | 4 | 87 | Over | Com\_5716\_neg |
| map04713 | Circadian entrainment | 0.0459770114942529 | 0.0715197956577267 | 1 | 1 | 4 | 87 | Over | Com\_6399\_neg |
| map00400 | Phenylalanine, tyrosine and tryptophan biosynthesis | 0.0903501737503341 | 0.126490243250468 | 1 | 2 | 4 | 87 | Over | Com\_1053\_neg |
| map04080 | Neuroactive ligand-receptor interaction | 0.214221695093434 | 0.271979447506595 | 1 | 5 | 4 | 87 | Over | Com\_6399\_neg |
| map00230 | Purine metabolism | 0.252552344113267 | 0.271979447506595 | 1 | 6 | 4 | 87 | Over | Com\_5716\_neg |
| map00360 | Phenylalanine metabolism | 0.252552344113267 | 0.271979447506595 | 1 | 6 | 4 | 87 | Over | Com\_2139\_neg |
